# An Oxygen-Releasing Mouthwash Reduces *Porphyromonas gingivalis* Biofilm and Suppresses *fimA* and *hagA* Expression

**DOI:** 10.1016/j.identj.2026.109432

**Published:** 2026-02-10

**Authors:** Fatemah M. AlAhmari, Rhodanne Nicole A. Lambarte, Terrence S. Sumague, Mary Grace B. Vigilla, Marwa Y. Shaheen, Sumaiah Ajlan, Lamees R. Alssum, Amani M. Basudan, Abdurahman A. Niazy

**Affiliations:** a Department of Periodontics and Community Dentistry, College of Dentistry, King Saud University, Riyadh, Kingdom of Saudi Arabia; b Molecular and Cell Biology Laboratory, Prince Naif bin AbdulAziz Health Research Center, King Saud University Medical City, Riyadh, Kingdom of Saudi Arabia; c Department of Oral Medicine and Diagnostic Sciences, College of Dentistry, King Saud University, Riyadh, Kingdom of Saudi Arabia

**Keywords:** *Porphyromonas gingivalis*, Oxygen-releasing mouthwash, Biofilm inhibition, Virulence, Chlorhexidine, Cytotoxicity

## Abstract

**Introduction and Aims:**

*Porphyromonas gingivalis* is a key periodontal pathogen whose biofilm and virulence limit the effectiveness of mouthwashes such as chlorhexidine (CHX). This study evaluated the antibacterial, antibiofilm and virulence effects of an oxygen-releasing mouthwash against *P. gingivalis*, complemented by in silico docking and cytotoxicity testing on human oral fibroblasts.

**Methods:**

*P. gingivalis* ATCC 33277 was grown anaerobically. Minimum inhibitory (MIC), bactericidal (MBC) and biofilm inhibitory concentrations (MBIC) were determined by resazurin-based microdilution and crystal violet biofilm assays, with 0.12% CHX as control. Biofilm structure and viability were analysed by confocal microscopy. Quantitative PCR measured expression of 6 virulence- and biofilm-associated genes. Molecular docking of sodium perborate to protein targets used AutoDock GNINA. Fibroblast cytotoxicity (ISO 10993-5 threshold ≥70% viability) was evaluated over 30 min to 24 h.

**Results:**

Bluem mouthwash showed concentration-dependent inhibition of *P. gingivalis* with minimum inhibitory (MIC), bactericidal (MBC) and biofilm inhibitory concentrations (MBIC) values of 0.78%, 1.56% and 3.12%, respectively. At 0.78%, biofilm biomass decreased to around 45% and at concentrations of ≥3.12% was reduced to ≤20%, with greater reduction than 0.12% CHX. Confocal imaging showed reduced biomass and thickness with a predominance of nonviable cells at higher concentrations. Bluem mouthwash was associated with downregulation of virulence-related genes, with *fimA* and *hagA* expression reduced at concentrations ≥0.78%, whereas 0.12% CHX increased *fimA* expression. Molecular docking predicted moderate binding affinities of sodium perborate with key virulence proteins, including kgp and mfa1. Bluem concentrations ≤0.78% maintained noncytotoxic fibroblast viability, while ≥1.56% and 0.12% CHX were cytotoxic across all tested periods.

**Conclusion:**

The oxygen-releasing mouthwash inhibited *P. gingivalis* biofilm formation, modulated virulence-associated gene expression and showed a wider noncytotoxic range on oral periodontal fibroblasts than chlorhexidine.

**Clinical Relevance:**

These findings on an oxygen-releasing mouthwash may help guide concentration selection and inform its potential adjunctive use in periodontal protocols, but clinical outcome studies are still needed before routine use can be recommended.

## Introduction

Periodontitis is the most common chronic inflammatory condition, characterised by progressive inflammation that compromises the periodontal complex structure, leading to irreversible severe damage to the bone and tissue supporting the tooth if not treated effectively.^1^ Its high prevalence as an oral disease affects nearly half of the global population, is associated with multiple systemic comorbidities and represents a significant public health concern.^2^^,^^3^

*Porphyromonas gingivalis*, an anaerobic gram-negative bacterium, is a key pathogen in the initiation and progression of chronic periodontitis.^4^ This is attributed to its capacity to express virulence factors involved in bacterial adhesion, disruption of the host immune response and gingival tissue damage.^5^^,^^6^ Moreover, the capacity to form biofilms further enhances antimicrobial treatment tolerance and aggravates periodontal diseases.^7^, ^8^, ^9^

Chlorhexidine digluconate (CHX) is widely used as a clinical gold standard for the treatment of periodontal disease because of its broad-spectrum antimicrobial activity. As a cationic biguanide, CHX is also a common agent used in surgical procedures and mouth-rinse products against periodontal pathogens by causing cell wall adsorption, resulting in bacterial cytoplasmic precipitation and cell death.^10^, ^11^, ^12^ Although CHX has high substantivity for reducing oral bacterial infection, it also alters the oral microflora, resulting in microbial dysbiosis.^13^^,^^14^ Importantly, a related study demonstrated the disruption of *P. gingivalis* biofilm-forming cells; however, it exhibited limited efficacy against disrupted cells and residual biofilms persisted, serving as scaffolds for re-colonisation.^15^

Due to the adverse effects of CHX and its limitations against mature biofilms, there is growing interest in alternative approaches that either modulate or debulk established biofilms while preserving host-cell compatibility rather than complete oral bacteria eradication. A recent study in multispecies oral biofilm models have shown that chemical interventions can regulate biofilm development and behaviour, underscoring the value of antibiofilm strategies that target biofilm phenotypes rather than planktonic susceptibility alone.^16^

Oxygen-releasing oral care products, such as peroxygen-based formulations, have emerged as clinically relevant adjuncts to ensure safer and more effective oral healthcare management, particularly for anaerobe-rich niches where oxygen tension declines with pocket depth. Bluem mouthwash (O_2_MW) is a commercially available, active-oxygenating formulation developed for daily dental routine, adjunct treatment of oral-related problems and support of wound healing.^17^ Its primary oxygen-releasing and antimicrobial ingredient is sodium perborate (SP), which generates reactive oxygen species in aqueous conditions; while the product also contains additional components (honey, xylitol, lactoferrin and other natural active ingredients) intended to support oral health.^18^ Several studies have reported significant gingival improvements and efficient plaque reduction compared with those of CHX.^19^^,^^20^ Furthermore, a recent study showed that Bluem has strong antibiofilm activity against *Fusobacterium nucleatum* and *Enterococcus faecalis*, with better efficacy than calcium hydroxide in an ex vivo model.^21^ Consequently, sodium perborate was considered the principal active component of interest in the present study.

Despite increasing clinical interest, limited data are available on the molecular mechanisms by which oxygen-releasing mouthwashes affect *P. gingivalis* biofilm formation and virulence. The expression of several virulence-associated key genes play a critical role in the pathogenesis of chronic periodontitis.^22^ These include *fimA* and *mfa1,* which encode the major and minor fimbrial structural subunit proteins responsible for regulating bacterial adhesion, biofilm formation and invasion of oral cavity tissue.^23^^,^^24^ Lys-gingipain (*kgp)* and Arg-gingipains A and B (*rgpA* and *rgpB)* are the 3 major cysteine proteases that modulate host inflammatory responses, induce tissue destruction and contribute to pathogenicity.^25^, ^26^, ^27^ Moreover, hemagglutinin A (*hagA*) mediates hemagglutination and hemoglobin binding to facilitate heme acquisition and gingipain maturation.^28^^,^^29^

This study aimed to investigate the antibacterial and antibiofilm effects of Bluem oxygenating mouthwash against *P. gingivalis* in vitro, as well as to analyse changes in the expression of key virulence genes, complemented by molecular docking of sodium perborate (as the oxygen-releasing active ingredient) to selected *P. gingivalis* virulence targets and assess potential cytotoxicity in oral periodontal fibroblasts.

## Materials and methods

### Bacterial strain and culture conditions

*P. gingivalis* derived from ATCC 33277 (Microbiologics, Kwik-stik, 0912) was activated and grown on a Brucella blood agar supplemented with hemin and vitamin K (Watin Biolife) according to the manufacturer’s instructions.^30^ The agar plate was placed onto a maintained atmosphere using an anaerobic jar (Thermo Scientific, Oxoid Anaerojar, Basingstoke) and gas-generating sachets (Thermo Scientific, Oxoid AnaeroGen 2.5 L, Basingstoke) and incubated for 5 days at 37°C. A modified tryptic soy broth (mTSB) (ATCC Medium 2722) was prepared for all assays. All bacterial broths were incubated in an Excella E24 incubator shaker (New Brunswick Scientific) at 37°C and 140 rpm.

### Turbidometric MIC and MBC determination assay

Commercially available Bluem mouthwash (BlueM Europe) was diluted in fresh mTSB medium to achieve 12 two-fold serial dilutions ranging from 100% to 0.004% in a 96-well round-bottom plate (Greiner Bio-One GmbH) and the effect of the oxygen-releasing oral care wash against *P. gingivalis* was assessed for minimum inhibitory concentration (MIC) determination. CHX mouthwash (0.20%, Parodontax Extra, Omega Pharma Manufacturing GmbH) was diluted in mTSB to a final concentration of 0.12% immediately prior to use. Diluted chlorhexidine was added as the positive control and mTSB alone served as the broth sterility control. Precise broth microdilution was conducted in accordance with the Clinical and Laboratory Standards Institute (CLSI) protocol.^31^ Approximately 50 µL of *P. gingivalis* bacterial suspension in mTSB at 0.08 A (OD600) was added to the wells and incubated for 24 h at 37°C in an anaerobic environment. After incubation, a working solution of 20 µL of 0.015% resazurin (Sigma-Aldrich) was added to all wells and incubated for 3 h at 37°C. The MIC was determined through visual assessment of the columns wherein the resazurin dye colour remained unchanged were considered to be at or above the MIC. Fluorescence measurements were recorded using a microplate reader (Synergy HT, BioTek Instruments) at excitation and emission wavelengths of 530 and 590 nm, respectively. The minimum bactericidal concentration (MBC) was determined by adding 10 µL of cell suspension to modified tryptic soy agar plates (mTSA; ATCC Medium 2722) starting from the MIC to the highest concentration, followed by anaerobic incubation for 24 h. Subsequently, the MBC was determined by visually assessing the agar plates with no growth of colonies.

### Biofilm inhibition assay

The effects on *P. gingivalis* of biofilm biomass formation after O_2_MW exposure were assessed using a microtiter assay in 96-well U-shaped bottom plates. Bacterial cell counts were standardised using a spectrophotometer (Libra S22, Biochrom Ltd.) and inoculated in mTSB at a standardised turbidity of 0.08 A (OD600). Aliquots were dispensed into plates and incubated anaerobically at 37°C for 72 h in the presence or absence of O_2_MW to allow formation of mature biofilms, consistent with established biofilm models to evaluate susceptibility of developed biofilms rather than early adhesion.^32^, ^33^, ^34^ After incubation, the media were discarded, the plate was washed gently with sterile distilled water and dried for 20 min. Each well was stained with 150 µL of 0.5% crystal violet solution (Sigma-Aldrich) for 10 min at room temperature. Subsequently, 200 µL of absolute ethanol (Sigma-Aldrich) was added to each well and incubated for 15 min at room temperature. The dye intensities in the samples were quantified at 490 nm using a microplate reader (Biotek Instruments, Synergy HT).

### CLSM analysis of biofilm eradication

The effects of O_2_MW on preformed biofilms of *P. gingivalis* were assessed using confocal laser scanning microscopy (Nikon C2; Nikon Instruments Inc.). After biofilms were allowed to form on sterile coverslips, the preformed samples were treated with O_2_MW or CHX for 5 min. Following treatment, all samples were carefully transferred to 6-well plates and gently washed with phosphate-buffered saline (PBS) to remove residual mouthwash and detached cells. Approximately 200 µL of LIVE/DEAD BacLight (Invitrogen Ltd.) working solution was used to stain the entire surface of the coverslip, followed by incubation in the dark at room temperature for 30 min, according to the manufacturer’s instructions. SYTO 9 (BacLight Component A) was illuminated using a 488 nm laser and the emission was recorded through a 520 to 550 nm bandpass filter (green channel). The fluorescence of propidium iodide (BacLight Component B) was excited using a 568 nm laser and detected using a 600 to 630 nm filter (red channel). Three-dimensional structures of the treated and untreated biofilms were constructed and visualised using NIS-Elements Advanced Research Software (version 4.0). Finally, the generated confocal z-stacks were analysed using COMSTAT2 (version 2.1) to evaluate the effect of oxygenating agent on residual *P. gingivalis* biofilm biomass and thickness structure.^35^^,^^36^

### Quantitative PCR analysis

The effect of O_2_MW on the gene expression of selected *P. gingivalis* targets was carried out using quantitative real-time PCR. Six *P. gingivalis* genes associated with biofilm formation and virulence were selected in this study, as previously described.^37^ The *P. gingivalis* inoculum was standardised to 0.05 A (OD600) and incubated in a 6-well plate with 1 mL of mTSB for 1 and 6 h exposure containing O_2_MW or CHX concentrations. After incubation, the cells were collected and transferred to an Eppendorf tube and centrifuged for 5 min at 10,053 × g. After pellet collection, total RNA extraction (BioFACT, Total RNA Prep Kit Ver.2.0, RP101-100, Yuseong-gu, Daejeon, Korea) was performed following the manufacturer’s instructions. The quality and concentrations of the extracted total bacterial RNA were measured using a BioSpectrometer basic (Eppendorf). The cDNA was obtained by reverse transcription using RT Ace First Strand cDNA synthesis kit (Haven Scientific) and a GeneAmp PCR System 9700 thermal cycler (Applied Biosystems), according to the manufacturer’s instructions. The PCR primers for 6 and reference genes are shown in Supplementary Table 1 (Haven Scientific) were added to the master mix solution EverGreen Universal Real-time PCR Master Mix (Haven Scientific) and mixed with the cDNA samples in 96-well PCR plate (MicroAmp EnduraPlate 96-Well Clear Reaction Plate; Applied Biosystems). qPCR was performed using the ABI 7500 Real-Time PCR System (Applied Biosystems) under the reaction conditions recommended by the manufacturer. Gene expression was normalised using *16S rRNA* as a reference gene and relative fold changes were calculated using the 2^−ΔΔCT^ method.^38^ All samples were analysed in triplicate using different RNA samples from 3 independent experiments.

### Molecular docking study

Molecular docking was performed against selected *P. gingivalis* protein targets associated with biofilm adhesion and gingipain-mediated proteolysis, which are commonly investigated in experimental studies of this keystone periodontal pathogen, to explore the potential interactions of O_2_MW. The crystal structures of *P. gingivalis* fimbrilin (fimA, PDB ID: 4Q98), gingipain K (kgp, PDB ID: 6I9A), fimbrial shaft protein (mfa1, PDB ID: 5NF2) and arg-specific cysteine proteinase gingipain R (rgpB, PDB ID: 1CVR) were obtained from the RSCB Protein Data Bank (RSCB PDB).^39^ For hemagglutinin A (hagA) and arginine-specific gingipain A (rgpA), the amino acid sequences were obtained from the UniProt database^40^ and the 3D protein structures were obtained using AlphaFold.^41^ Protein structure preparation was carried out through the Protein Repair and Analysis Server (PRAS)^42^ and AutoDock tool protocol.^43^ Sodium perborate was selected as the candidate ligand for this study, which is the primary oxygen-releasing component present in the O_2_MW product, as previously described.^18^ The SMILES format (Sigma-Aldrich; BNaO_3_·4H_2_O; CAS: 10486-00-7) was generated using ChemAxon-Marvin JS (version 22.11.1; www.rcsb.org/chemical-sketch), the sodium ions were removed to eliminate artifacts in the docking calculation,^44^ and the final structure was exported to sdf file. Subsequently, the 3-dimension structure was rendered and optimised using Avogadro software (version 1.2.0) and exported to mol2 format.^45^

AutoDock Tools software (version 1.5.7)^46^ was used to generate ligand and receptor pdbqt files for the molecular docking simulations. Blind docking was carried out using GNINA software (version 1.3.2)^47^ to identify favorable ligand conformations across the entire receptor. The search space definition was referenced using the whole receptor and padded by 4 Å. To ensure thorough sampling of the ligand search space, the exhaustiveness was set to 64 and the number of output poses to 20 and the CNN re-scoring function was utilised for final ranking. The first top-ranked docking pose was selected for analysis and visualisation. The ligand-receptor complex was computed using PyMOL software (version 3.0.3), visualisation of the 3D complex conformations were rendered using ChimeraX software (version 1.7.1) and protein-ligand interactions analysis were analysed by LigPlot+ software (version 2.3.1).^48^

### Cytotoxicity

Human periodontal ligament fibroblasts (HPLFs; Cat. No. 2630; ScienCell Research Laboratories) were cultured in complete growth medium under aseptic conditions in a laminar flow hood. Cells were maintained at 37°C in a humidified atmosphere of 5% CO₂ and 95% humidity until reaching ∼80% confluency. Upon confluence, the cells were detached and seeded into 96-well culture plates at a density of 1 × 10⁴ cells/well in 100 μL. After 24 h, the cells were treated with the indicated concentrations of O_2_MW for 1 min at 37°C, after which the treatment medium was replaced with fresh DMEM.

Cell viability was evaluated using the AlamarBlue reagent (Invitrogen) according to the manufacturer’s instructions. At specified time points (30 min, 1 h, 6 h and 24 h post-treatment), 10 μL of AlamarBlue substrate was added to each well and plates were incubated in the dark at 37°C for 4 h. Background fluorescence was corrected using blank wells containing culture medium and reagent. Fluorescence was measured using a BioTek Synergy HT microplate reader at λexc 530 nm and λem 590 nm and the results were expressed as the percentages of metabolically active viable cells.

### Statistical analysis

Data obtained from 3 independent experiments performed in triplicate are presented as the mean ± standard deviation (SD). GraphPad Prism (version 6.00 for Windows; https://www.graphpad.com) was used for all statistical analyses and graph generation. Based on the data, 1-way analysis of variance (ANOVA) or two-way ANOVA was used to evaluate significance, followed by either Dunnett’s or Tukey’s post hoc test analysis. Differences were considered statistically significant at *P* ≤ .05.

## Results

### MIC and MBC Determination

**Fig. 1:**
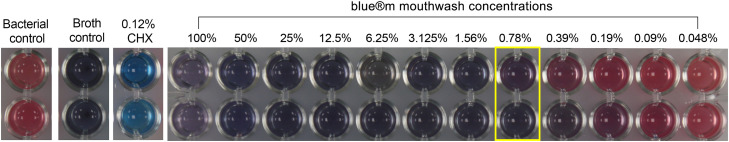
Resazurin-based MIC determination of O_2_MW against *P. gingivalis*.

Microdilution assays demonstrated a clear concentration-dependent inhibition of *P. gingivalis* by O_2_MW (Figure 1). The minimum inhibitory concentration (MIC) was identified at 0.78%, where duplicate wells remained bluish purple indicating absent metabolic activity, while growth resumed at ≤0.39% (pinkish red wells). The antibacterial positive control (0.12% chlorhexidine) showed complete inhibition, whereas the bacterial growth control exhibited the expected colour shift indicative of active metabolism; the broth-only control remained unchanged. The MIC and MBC values for O_2_MW tested in *P. gingivalis* are shown in Table 1.

**Table 1:** MIC and MBC values of O_2_MW tested concentrations.

MIC	MBC	MBIC
0.78%	1.56%	3.12%

### Biofilm biomass quantification

**Fig. 2:**
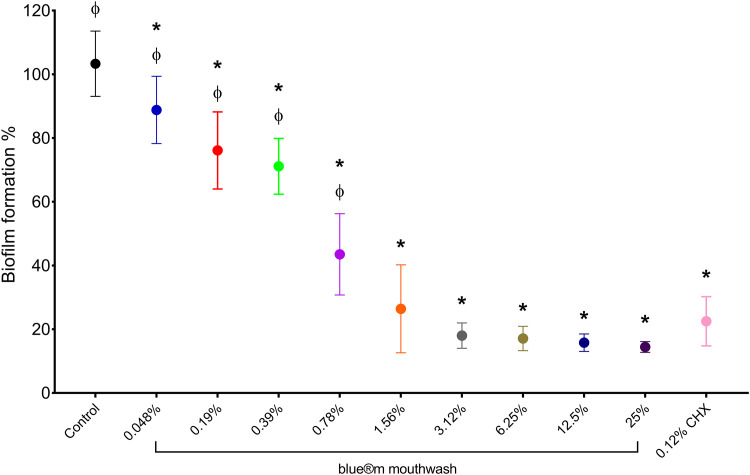
Effects of the different concentrations of O_2_MW on the biofilm formation of *P. gingivalis*. Data are expressed as mean ± SD (n = 9); Asterisk (*) indicates *P* < .05 compared with control and Phi (Ф) indicates *P* < .05 compared with 0.12% CHX.

O_2_MW significantly reduced *P. gingivalis* biofilm formation in a concentration-dependent manner (Figure 2). Relative to the untreated control (100%), biofilm biomass decrease was observed at 0.048% to 0.39% (90%-70%), followed by a significant reduction at 0.78% (45%). Biofilm biomass dropped to 25% to 30% at 1.56% and reached 15% to 20% at ≥3.12%, with no further decrease up to 25%. The 0.12% CHX yielded 20% to 25%. Thus, O_2_MW at ≥3.12% achieved biofilm inhibition greater than CHX, while lower concentrations produced incomplete biofilm suppression.

### Confocal live/dead imaging

**Fig. 3:**
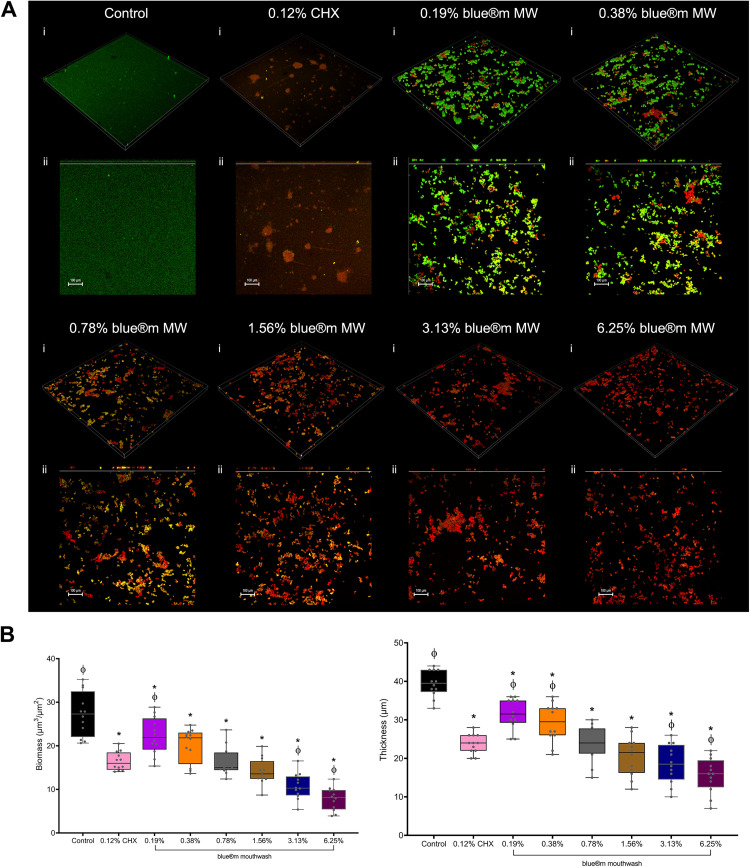
(A) Representative CLSM biofilm images of *P. gingivalis*. (i) three-dimension and (ii) thickness and top view z-projection. Biofilms of untreated control and treatment with 0.19, 0.38, 0.78, 1.56, 3.13 and 6.25% of O_2_MW; and 0.12% CHX. Biofilm measurement of (B) biomass (µm^3^/µm^2^) and (C) thickness (µm^2^). Data are expressed as mean ± SD (*n = 9*); Asterisk (*) indicates *P* < .05 compared with bacterial control and phi (Ф) indicates *P* < .05 compared with CHX. Magnification: ×20; scale bar: 100 µm.

Representative CLSM micrographs (Figure 3) showed dense, confluent *P. gingivalis* biofilms in the untreated control, dominated by green (viable) fluorescence, with compact microcolonies and minimal red (nonviable) signal. Sub-inhibitory O_2_MW concentrations (≤0.39%) produced partial disruption, with mixed viable fluorescent signals and scattered red zones. At 0.78% (planktonic MIC), the biofilm architecture became visibly porous and with fragmented clusters, showing increased red signals indicative of membrane compromise. At ≥3.12%, residual biomass appeared to be thin, predominantly red with small, aggregates; similar to 0.12% CHX. Biofilm thickness reduced around 0.78% to 3.12% treatment and reached lower levels at higher concentrations.

### qPCR analysis of virulence gene expression

**Fig. 4:**
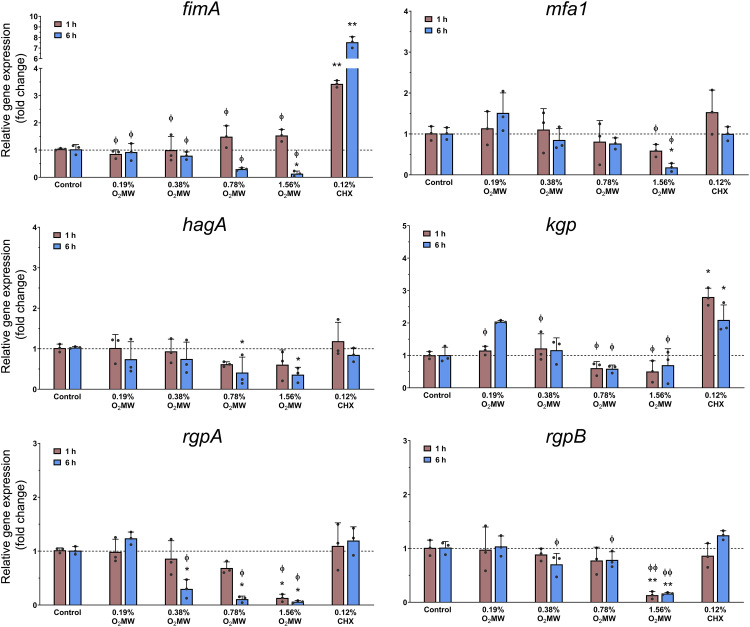
Relative *fimA, mfa1, hagA, rgpA, rgpB,* and *kgp* expression (fold change) after exposure with O_2_MW. Asterisk indicates (*) *P* < .05 and (**) *P* < .01 compared with bacterial control. Phi indicates (Ф) *P* < .05 and (Ф Ф) *P* < .01 compared with 0.12% CHX.

Quantitative PCR showed a dose-dependent suppression of *P. gingivalis* virulence-associated genes by O_2_MW treatment (Figure 4). Relative to the untreated control, *hagA* expression was only modestly reduced at ≤0.39% (0.7-0.9-fold), dropped sharply at 0.78% (0.4-fold) and reached a decreased fold change at 1.56% (0.2-fold), relative to 0.12% chlorhexidine (CHX). *fimA* followed a similar pattern, minimal change at ≤0.39%, pronounced down-regulation at ≥0.78% (0.5-fold). While for CHX treatment, expression levels were increased (from 3-fold to 8-fold change). These data indicate that O_2_MW down-regulates key adhesion and hemagglutinin genes (*fimA, hagA*) at sub-inhibitory concentrations, with 0.78% (MIC) producing transcriptional suppression relative to chlorhexidine.

### Molecular docking studies

**Fig. 5:**
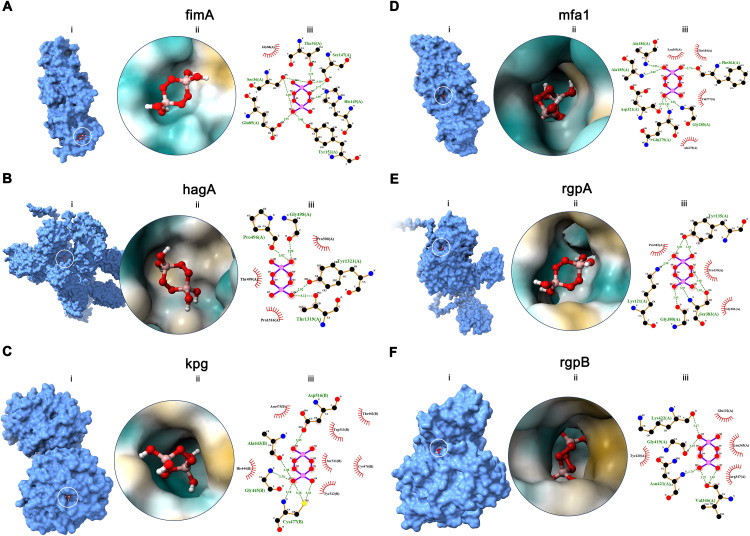
Molecular-docked complexes of (A) fimA, (B) hagA (C) kpg, (D) mfa1, (E) rgpA, and (F) rgpA with ligand. Panel represents (i) surface representation showing the ligand docked onto the receptor pocket; (ii) three-dimension hydrophobic surface interaction view illustrating the receptor residues hydrogen bonds; and (iii) interaction diagram of receptor-ligand complex depicting the hydrogen bonding interactions (green dash line).

Molecular docking studies of 6 *P. gingivalis* proteins with SP (O_2_MW oxygen-releasing component), as shown in Figure 5, were performed to explore potential interactions of SP on bacterial adhesion- and virulence-related proteins. Binding affinity scores ranged from −5.39 to −6.19 kcal/mol; detailed results are outlined in Supplementary Table 2. Moderate predicted binding score were observed for kgp (−6.19 kcal/mol), mfa1 (−6.19 kcal/mol), fimA (−5.84 kcal/mol) and rgpA (−5.88 kcal/mol), each with more than 3 hydrogen bonds, whereas low-to-moderate binding affinities were predicted in hagA (−5.39 kcal/mol) and rgpB (−5.59 kcal/mol) also showing more than 3 hydrogen-bond interactions. Furthermore, predicted contact residues for SP included kgp (ALA443, ASP516, CYS477 and GLY445 ranging 2.85-3.35 Å distance); mfa1 (ALA186, ALA185, ASP321, GLN279, GLY280 and PHE364 ranging 2.51-3.03 Å distance); rgpA (TYR135, TYR135, LYS121, GLY380, SER383, SER383 and SER383 ranging 2.85-3.08 Å distance); fimA (GLU85, HIS149, SER36, SER36, SER147, SER147, THR34 and TYR152 ranging 2.70-3.08 Å distance); hagA (GLY498, PRO496, THR1319 and TYR1321 ranging 2.92-3.12 Å distance); and rgpB (LYS422, GLY419, ASN421, VAL346 and VAL346 ranging 2.75-3.27 Å distance). Overall, a total of 36 contacts, 28 unique residues and an average contact distance ranging from 2.77 to 3.16 Å were predicted.

### Cytotoxicity

**Fig. 6:**
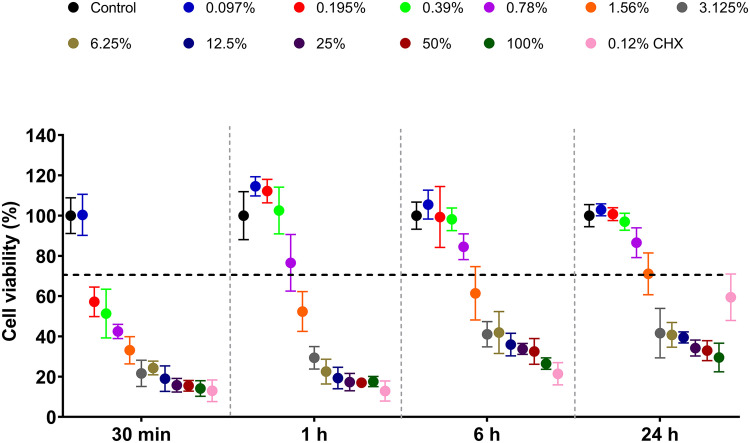
Human fibroblasts cell viability from 30 min to 24 h exposure with 0.097% to 100% O_2_MW and 0.12% CHX treatment. Data are presented as mean cell viability percentages ± SD (*n = 6*) at each time point.

Human oral periodontal fibroblasts exposed to O_2_MW showed a clear dose and time-response profile across all time points (Figure 6). At 30 min, concentrations ≤0.39% maintained viability at or above the noncytotoxic threshold, whereas 0.78% trended toward the 70% line and ≥1.56% reduced viability well below 50%. Concentrations ≤0.78% consistently met the ≥70% viability benchmark (ISO 10993-5), with ≤0.39% occasionally showed slight increases at early time points. In contrast, 0.12% chlorhexidine (CHX) suppressed viability at every interval and did not achieve the noncytotoxic threshold by 24 h. O_2_MW concentrations ≥1.56% were cytotoxic, whereas the sub-percent range (≤0.78%) remained biocompatible over 24 h. Overall, O_2_MW demonstrated a wider noncytotoxic window than CHX for human oral fibroblasts.

## Discussion

This in vitro study demonstrates that an oxygen-releasing mouthwash (O_2_MW) reduces *P. gingivalis* activity based on growth inhibition (MIC = 0.78% v/v), biofilm biomass reduction with architectural collapse and transcriptional downregulation of 6 key virulence genes: namely *fimA, mfa1, hagA, rgpA, rgpB* and *kgp*. Collectively, these effects showed chlorhexidine-comparable treatment at ≥3.12%, with potential impacts on both aggregation and protease-related processes, which are central to *P. gingivalis* pathogenicity.^49^, ^50^, ^51^, ^52^, ^53^, ^54^

Confocal laser scanning microscopy showed dose-dependent loss of viable architecture at the planktonic MIC and a transition to sparse, predominantly nonviable biomass at ≥3.12%, consistent with the crystal violet data. Biofilms were incubated for 72 h to allow development of mature and reproducible *P. gingivalis* biofilms in the static microtiter system, consistent with published *P. gingivalis* biofilm protocols for biomass and architecture assessment.^33^^,^^34^^,^^55^ Because biofilms exhibit inherent antimicrobial tolerance due to diffusion limitations, altered physiology and persister formation, observing CHX-like outcomes at higher O_2_MW concentrations highlights the potential of an oxygen-releasing mouthwash to reduce biomass and disrupt anaerobe-rich biofilms in vitro with adequate exposure.^56^, ^57^, ^58^ Previous experimental and clinical studies on hydrogen peroxide-based rinses support this biological plausibility.^59^, ^60^, ^61^

Oxygen tension declines with pocket depth, favouring obligate anaerobes such as *P. gingivalis* and reinforcing dysbiosis.^62^^,^^63^ By releasing oxygen, peroxygens may counter local hypoxia and impair the biofilm environment differently.^58^, ^59^, ^60^^,^^62^ Many *P. gingivalis* surface factors, such as gingipains, adhesins and hemagglutinins, are exported via the type IX secretion system (T9SS).^63^ In this study, the observed downregulation of *rgpA, rgpB, kgp, hagA, fimA* and *mfa1* may indicate effects on adhesion and potential impact on T9SS-dependent proteolysis, both central to nutrient capture, immune evasion and biofilm maturation.^2^^,^^63^^,^^64^

Specifically, downregulation of *fimA* and *mfa1* may impair early colonisation and biofilm stability, while reduced *hagA* expression could limit hemagglutination and heme-harvesting potential.^49^^,^^50^ In addition, decreased *rgpA, rgpB* and *kgp* expression is notable because gingipains regulate multiple virulence functions, such as extracellular matrix degradation and post-translational secretion of surface proteins, potentially contributing to reduced biofilm biomass.^51^^,^^52^ Previous studies indicate that *P. gingivalis* modulates virulence gene expression within the initial hours of contact with host epithelial or fibroblast cells, reflecting early signaling and adhesion events, supporting the relevance of the exposure timepoints in this study.^8^^,^^9^

These results are consistent with previous studies that shorter CHX exposures cause strong initial killing followed by rapid regrowth and metabolic shifts; reflecting stress-induced adaptation, likely explaining *fimA* upregulation during recovery.^65^ As a membrane-active cationic antiseptic, CHX has been associated with adaptive responses and reduced susceptibility in oral bacteria.^66^ In structured biofilms, diffusion limitations can create sublethal exposure gradients, enabling possible recolonisation once CHX concentrations drop below inhibitory levels.^61^ Under these conditions, increased CHX-associated upregulation of *fimA* expression may facilitate biofilm reattachment and reassembly, with potential clinical implications.^49^^,^^64^ If residual matrix or biofilm scaffolds persist after exposure, enhanced fimbrial expression could support attachment to host substrates, co-aggregation during early recolonisation and faster recovery of biofilm structure during the washout phase. This is consistent to illustrate that CHX can reduce viability yet fail to completely disrupt *P. gingivalis* biofilms.^15^ More broadly, antiseptic stress can reshape biofilm behaviour and recovery dynamics, highlighting concerns regarding stress-induced mechanisms during repeated CHX use.^61^^,^^66^ Together, these findings suggest that CHX could induce compensatory adhesion processes in surviving bacteria, which warrants targeted functional validation.

In *P. gingivalis*, fimbriae and gingipains are regulated under stress and do not directly correlate with binding affinity; binding strength does not guarantee downregulation, which helps explain why O_2_MW can outperform CHX for *mfa1* and *rgpA* at 6 h, while CHX remains stronger for *kgp*.^67^

Moreover, an in silico molecular docking analysis of the oxygen-releasing component of O_2_MW (SP) was performed against selected *P. gingivalis* adhesion factors (fimA and mfa1), proteolytic enzymes (kgp, rgpA and rgpB) and hemagglutinin (hagA) to identify potential interaction sites relevant to periodontal pathogenesis. The predicted binding affinities (−5.39 to −6.19 kcal/mol) suggest moderate interaction potential across several targets, which may help interpret impaired biofilm development or reduced protease-linked pathogenic functions, consistent with observed suppression of biofilm biomass and transcriptional programs.^68^, ^69^, ^70^ Notably, the observed downregulation and moderate interaction predicted of mfa1 and kgp, crucial virulence factors of *P. gingivalis* for surface attachment and biofilm formation, suggests the potential effects of oxygenating-agent in modulating co-aggregation with predominant oral bacterial flora on host molecule which may prevent the initiation of periodontal tissue infections.^23^^,^^24^ However, these docking results presented as exploratory, hypothesis-generating evidence and do not demonstrate inhibition, target engagement or biological activity and they should be interpreted as a basis for future biochemical validation in relevant in vivo and ex vivo models.

While CHX is a potent benchmark, multiple studies have also documented concentration- and time-dependent cytotoxicity that compromises fibroblast/keratinocyte viability and migration.^17^^,^^71^^,^^72^ Preliminary comparative in vitro data suggest that O_2_MW may be less cytotoxic than CHX in oral cell lines under matched exposures, indicating a potentially wider therapeutic window that warrants confirmation across oral cell types and exposure times.^73^ The results indicate that low-dose O_2_MW (≤0.78%) remained within the noncytotoxic range up to 24 h, whereas 0.12% CHX did not, despite its clinical antiplaque effectiveness. This aligns with the ISO 10993-5 convention and previous literature, wherein CHX, even at clinically used concentrations, compromises oral cell viability and related wound-healing functions in vitro.^74^, ^75^, ^76^, ^77^ A recent study suggests that oxygen-releasing formulations can preserve or enhance viability at low concentrations and may modulate inflammatory signaling.^19^^,^^20^^,^^78^

While in vitro cytotoxicity does not alone determine clinical performance, these findings support considering oxygen-releasing rinses such as O_2_MW as an adjunct where soft-tissue compatibility is critical. Further confirmation with standardised ISO-based cytotoxicity panels, including migration and collagen synthesis, as well as controlled clinical trials to define therapeutic margins relative to CHX, is needed.

This study has limitations, such as the monoculture *P. gingivalis* model and the absence of functional protease assays and residual-activity testing on tooth-like substrates. The full commercial formulation of Bluem mouthwash was evaluated to maximise clinical relevance, while in silico docking analyses focused on sodium perborate as a mechanistic proxy for the oxygen-releasing component. However, the observed effects cannot isolate the independent contribution of sodium perborate versus other components. Therefore, future work should include sodium perborate and appropriate control and fractionated formulations to determine the specific effects of each component on antibiofilm efficacy, virulence modulation and cytocompatibility.

Finally, oral biofilms are polymicrobial and interspecies interactions can reshape biofilm architecture and antimicrobial responses. Notably, recent work using controlled multispecies oral biofilm systems indicate that targeted chemical antibiofilm interventions can alter biofilm development beyond growth inhibition.^16^ This suggests that adjunctive agents may not only reduce biomass, but also shift less pathogenic phenotypes, which is important for translating single-species findings to complex periodontal communities.

Nevertheless, Bluem demonstrated CHX-comparable effects on *P. gingivalis* biofilms at concentrations ≥0.78% and downregulated key virulence genes encoding major fimbriae, hemagglutinin and gingipains. Given the central role of these factors in *P. gingivalis* virulence and biofilm persistence, these findings support the use of Bluem oxygen-releasing mouthwash as a plausible adjunctive agent against *P. gingivalis* and validate future studies in multispecies biofilm models and expanded host-compatibility testing before clinical translation.

## Author contributions

FMA, MYS, SA, LRA and AMB developed the study concept and design of the study, contributed to the analysis and interpretation, drafted and critically revised the manuscript. RNAL, TSS and AAN contributed to data acquisition, execution of the experiment, analysis, interpretation and critically revised the manuscript. MGV contributed to data acquisition, execution of the experiment and critically revised the manuscript. All authors gave their final approval and agree to be accountable for all aspects of the work.

## Funding

This research did not receive any specific grant from funding agencies in the public, commercial or not-for-profit sectors.

## Declaration of competing interest

None disclosed.

## Appendix Supplementary materials

Image, application 1
