# Supplementary material for: An Oxygen-Releasing Mouthwash Reduces Porphyromonas gingivalis Biofilm and Suppresses fimA and hagA Expression
Source: Int Dent J. 2026 Feb 10;76(2):109432. doi: 10.1016/j.identj.2026.109432 (PMC12914669; doi:10.1016/j.identj.2026.109432)
Supplement: Supplementary file 1 [file mmc1.docx]

**Supplementary Table 1.** Selected genes and primer sequences used in this study.

| **Gene** | **Primer Sequence (5′-3′)** |
| --- | --- |
| *fimA* | F: TGTTGGGACTTGCTGCTCTT  R: TTCGTCATCGCCAACTCCAA |
| *hagA* | F: CCGCGAGATTCTGGGCAATA  R: CCTGCTCCGATGAACTTGGT |
| *kgp* | F: GACCCTGCGTTGTAGCAGT  R: GGTGTTGCTAATGCCAGCG |
| *mfa1* | F: GATCCTGCAACCCACAATGC  R: AGCCTGAGCCTGAGTAGACA |
| *rgpA* | F: GTTCCATCACCGCTACCCAT  R: GGACAAGGACCGACGAAAGA |
| *rgpB* | F: CGTCTTGCCTTCAGTAGCGA  R: TGTAGAAAGTCCTGCTGCCG |
| *16S rRNA* | F: AGTCGCGTGAAGGAAGACTG  R: TACCGAACAACCTACGCACC |

**Supplementary Table 2.** Summary of the *P. gingivalis* receptors and SP complex GNINA predicted docking scores.

| **Receptor-ligand complex** | **Affinity (kcal/mol)** | **Total H-Bonds** | **Residue - Length (Å)** |
| --- | --- | --- | --- |
| fimA-SP | –5.84 | 8 | GLU85 – 2.91  HIS149 – 2.89  SER36 – 3.04  SER36 – 3.08  SER147 – 3.10  SER147 – 2.77  THR34 – 2.70  TYR152 – 3.08 |
| hagA-SP | –5.39 | 4 | GLY498 – 2.96  PRO496 – 3.07  THR1319 – 3.12  TYR1321 – 2.92 |
| kpg-SP | –6.19 | 6 | ALA443 – 2.85  ASP516 – 2.98  CYS477 – 3.19  CYS477 – 3.28  CYS477 – 3.26  GLY445 – 3.35 |
| mfa1-SP | –6.19 | 6 | ALA186 – 2.93  ALA185 – 3.03  ASP321 – 2.52  GLN279 – 2.93  GLY280 – 2.91  PHE364 – 2.71 |
| rgpA-SP | –5.88 | 7 | TYR135 – 3.08  TYR135 – 2.94  LYS121 – 2.98  GLY380 – 2.95  SER383 – 3.07  SER383 – 2.85  SER383 – 3.08 |
| rgpB-SP | –5.59 | 5 | LYS422 – 3.27  GLY419 – 3.10  ASN421 – 2.79  VAL346 – 2.75  VAL346 – 2.85 |
